# Factors Associated with the Maintenance of Breastfeeding at One Year among Women in Chiang Mai, Thailand

**DOI:** 10.3390/ijerph18179224

**Published:** 2021-09-01

**Authors:** Krongporn Ongprasert, Penprapa Siviroj

**Affiliations:** Department of Community Medicine, Faculty of Medicine, Chiang Mai University, Chiang Mai 50200, Thailand; krongporn.o@cmu.ac.th

**Keywords:** continued breastfeeding, determinants, Thailand

## Abstract

This study aimed to investigate factors associated with breastfeeding for at least one year among women in Chiang Mai, Thailand. We conducted a cross-sectional study of 451 mothers with children aged between 12 and 24 months who visited the well-baby clinic among women who visited the well-baby clinic in secondary and tertiary hospitals. The data collected included maternal sociodemographic information, employment status, reasons contributing to continued breastfeeding, primary sources of information, and influential people affecting continued breastfeeding. Multivariable logistic regression analysis was used to investigate the relationship between explanatory variables and continued breastfeeding at one year. Reporting “easier to bond with baby” as a reason to continue breastfeeding (AOR 3.118, 95% CI: 2.022, 4.809) and multiparous status (AOR 1.588, 95% CI: 1.042, 2.420) were positive predictors of mothers who had breastfeeding at least one year postpartum while mothers with undergraduate education level (AOR 0.635, 95% CI: 0.404, 0.997) were more likely to discontinue breastfeeding. Our study highlighted that working mothers have lower odds of continued breastfeeding than stay-at-home mothers (SAHMs), which was found for work with day shifts (AOR 0.437, 95% CI: 0.261, 0.731), work with rotational shifts (AOR 0.481, 95% CI: 0.247, 0.934), and work from home jobs with a flexible schedule (AOR 0.439, 95% CI: 0.229, 0.838). These findings showed that both employment outside home and work from home were strong risk factors for discontinuing breastfeeding before 12 months. We suggest that a breastfeeding-friendly workplace policy is essential to enhance the continuance of breastfeeding. Additionally, working at home requires more research to explore breastfeeding barriers and establish more support strategies.

## 1. Introduction

Most major health organizations recommended that infants be exclusively breastfed for the first 6 months of life and that continued breastfeeding with appropriate complementary foods continue for at least 1 year or more [1,2]. Recent evidence confirms that breast milk contains sevderal nutrients, biologically active substances and components associated with the immune system and is acceptable for growing children for at least two years of life when breastfeeding is continued along with the introduction of complimentary food [3,4,5]. A longer duration of lactation was associated with additional child and maternal health benefits. An inverse relationship between breastfeeding duration and the risk of metabolic syndrome, breast cancer and ovarian cancer in lactating mothers was established in previous studies [6,7,8]. Children who are breastfed for longer periods have a lower risk of being overweight or obese, lower rates of infectious morbidities and mortality, and higher performance on intelligence tests than those who are breastfed for shorter periods [9,10,11]. In addition to health benefits, prolonging breastfeeding may provide significant economic benefits in reducing direct and indirect costs. For example, breastfeeding reduced costs associated with buying infant formula, and breast milk provides optimal nutrition, allowing children to achieve their full physical and cognitive development, which might reduce potential medical fees, including physician, hospital, and procedural fees, and may improve adult productivity [12,13]. Furthermore, infant formula feeding generates more adverse environmental consequences than breastfeeding, e.g., greenhouse gas, plastic, and other waste, via the animal agriculture systems and the dairy industry [14,15].

Although the advantages of breastfeeding over infant formulae feeding have been extensively documented and continued breastfeeding at 12 months is one of the core indicators for assessing global infant and young child feeding practices [16], in most countries of the world, prolonged breastfeeding rates are typically suboptimal and only slowly improving [10]. The prevalence is more widespread in low-income and lower-middle-income settings than in high-income countries, but the overall rates are lower than the optimal level [10]. Additionally, the progression rates remain far from the global target set by the World Health Organization (WHO) that 80% of children be breastfed until 1 year of age by 2030 [16]. In Thailand, projects to promote and support breastfeeding have been applied and scaled up nationwide since 1989. The main activities were promoting the Baby-Friendly Hospital Initiative, the Code of Marketing of Breastmilk Substitutes and related products, and legislation on maternity leave, which currently offers 98 days of maternity leave with full pay [17]. Despite these efforts, the rate of breastfeeding up to a 1 year remained low. The national surveys conducted in 2012 [18], 2015 [19], and 2019 [20] found that the prevalence of continued breastfeeding at one year was 32.4%, 33.3%, and 24.6%, respectively. 

Breastfeeding practices are influenced by a complex combination of numerous variables, including demographic factors, psychosocial attributes, health-care attributes, biomedical constraints, community and policy. The factors that affect exclusive breastfeeding for six months have been widely investigated; however, these results cannot be applied in an extended breastfeeding context. Previous studies affirmed changes in the factors associated with breastfeeding maintenance over time [21,22,23,24]. Furthermore, the information related to the factors associated with continued breastfeeding for 12 months is limited: the majority of recent studies were conducted in developed countries, such as the United States [23], Italy [25], Norway [26] and Australia [27,28,29], and only a few were conducted in developing countries [21,30,31]. The factors that influence breastfeeding maintenance for 12 months require more in-depth study in Thailand, a country that has been under investigated in the literature previously.

This study aimed to investigate factors associated with breastfeeding for at least one year among women in Chiang Mai, Thailand. The findings will help to identify potential factors that could indicate areas for further study and may help in the planning of health actions aimed at increasing the proportion of mothers who breastfeed for one year or more according to the international recommendation.

## 2. Materials and Methods

### 2.1. Setting

This study was carried out in four hospitals located in Chiang Mai municipality, Thailand: one private hospital and three public hospitals (secondary care and tertiary care level).

### 2.2. Participants

Sample size was calculated using EpiInfo^TM^ version 7.2 [32] based on the population survey or descriptive study. We used the population size (15,648 persons) from the number of newborns in Chaing Mai Province in 2018 [33]. The expected frequency (33.3%) was the prevalence of mothers breastfeeding at one year, in accordance with the previous values reported in Thailand Multiple Indicator Cluster Survey 2016 [19], the confidence level of 97%, an acceptable margin of error of 5%, and design effect of 1.0. Finally, the total sample size was determined to be 408; we aimed for 10% oversampling, so a minimum of 448 women was needed.

A total of 451 mothers with children aged 12–24 months visited the well-baby clinic in four hospitals on recruitment days from January 2019 to October 2019. The participants were randomly selected. Mothers were eligible if they were aged 18 years or over and had children without congenital disease or oral cavity malformations that could affect breastfeeding. In addition, mothers were excluded if they self-reported having a condition leading to the mother being unable to breastfeed, such as acquired immune deficiency syndrome or use of cancer chemotherapy medications. Before providing information, all participants signed informed consent forms. In this study, the mothers were categorized into two groups. The mothers who continued nursing the infant with breastmilk by any breastfeeding method (feeding directly at the breast, expressed breastmilk feeding, or mixed feeding) were defined as the success group, whereas the other group refers to those that did not reach the 12-month breastfeeding target.

### 2.3. Data Collection

We contacted the hospital directors for collaboration and permission to recruit participants. The interviews were performed in the waiting area through face-to-face interviews by three researchers until the final sample number was reached. Before beginning the interview, the researchers provided verbal instruction regarding the research’s overall concept, including the purposes and data collection process of the study. Those who agreed to participate were informed that the interview was anonymous, participation was voluntary, and no payment would be given. Before providing information, all participants answered a set of questions corresponding to the inclusion and exclusion criteria and signed informed consent forms. The questionnaire took approximately 15–20 min to complete.

### 2.4. Instrument

The items included in the questionnaire were chosen based on previous studies [34,35] that were considered interesting or that were meant to address the aims of the study by the research team. The questionnaire consisted of the following four sections:

(1) The demographics and characteristics of the mothers included maternal age, level of education, monthly income of the household, marital status, body mass index (BMI), parity, timing of first antenatal care (ANC), age of infant when mother returned to work, employment status, and shiftwork type. For the evaluation of shiftwork type, we categorized into four groups: stay-at-home mothers (SAHMs), full-time work with day shift, work with rotational shift, and work-from-home job with a flexible schedule. Full-time work with a day shift refers to the work that has a fixed schedule required to work from 8 a.m. to 8 p.m., for example, hairdresser, teacher, and mechanic. Work with rotational shift refers to work that has a consistent or predictable change in shifts each day or week, for example, nurses, policemen, and factory workers. Finally, work-from-home job with a flexible schedule refers to the work that mothers can do whenever they desire to work as long as the work is completed, for example, journalist, translator, and online seller.
(2) Reasons that encouraged the decision to breastfeed as identified by mothers who continued breastfeeding at one year postpartum in which reasons were measured by questions that required a nominal or categorical (yes or no) response, including better for child’s health, better for mother’s health, avoid trouble from formula feeding, easier to bond with baby if breastfeeding, more convenient than formula feeding, more cost-effective than formula feeding, family members or friends encourage breastfeeding, and healthcare providers encourage breastfeeding.
(3) Mother’s primary sources of information related to continued breastfeeding at one year postpartum in which the data were measured by questions that required nominal or categorical (yes or no) face-to-face communication with health care providers, prenatal class, television, internet/social media, and printed media.
(4) Influential persons’ impact on continued breastfeeding in which the data were measured by questions that required a nominal or categorical (yes or no) response including myself, husband, maternal grandmother, friends, nurses, and doctors.

The participants could check off all the choices that apply to them in sections two, three, and four. To validate the survey, enhance patient comprehension and avoid ambiguity, each question was extensively evaluated by one pediatrician and three lactation consultant nurses. Prior to conducting this investigation, a pilot study was carried out among a sample of 10 women (not included in the final sample) to evaluate the comprehensibility and validity of the questions.

### 2.5. Ethical Consideration

This study obtained approval from the Research Ethics Committee, Faculty of Medicine, Chiang Mai University (No. 158/2018). Written informed consent was obtained from all participants. This study complied with the principles set forth in the Declaration of Helsinki (1964) and all of its subsequent amendments.

### 2.6. Statistical Analysis

In this study, continuous variables are presented as the mean ± standard deviation (SD), and categorical data are presented as frequencies and percentages. The chi-square test was used to test the differences in categorical variables between the participants who succeeded and those that did not reach the 12-month breastfeeding target. A bar chart was used to display factors that encouraged them to breastfeed, type of mother’s primary sources of information related to continuing breastfeeding, and influential person that impacted the decision to continue breastfeeding at one year postpartum to show the comparisons of each category in a frequency distribution. Binary logistic regression was used to analyze all variables, including demographics and maternal characteristics, factors that encouraged the decision to breastfeed, the mother’s primary information sources and influential persons that impacted the breastfeeding decision. We did not include maternal BMI or marital status for logistic regression analysis because maternal BMI was the current BMI that probably changed from when the infant was one year old, and the proportion of mothers who were not married was low (2.9%). Additionally, prenatal classes always arrange with small groups and mothers can converse directly with health care providers that share characteristics with face-to-face communication. Thus, prenatal class was not included for analysis. Odds ratios (OR) and 95% confidence intervals (CI) were estimated using logistic regression with the forward stepwise method for selecting variables. The prediction model was analyzed with confounder adjustment including maternal education graduate level, multiparous status, shiftwork types and full-time work with a day shift, work with a rotational shift, and work-from-home jobs with a flexible schedule, and all these variables about reasons that encouraged the decision to breastfeed, mother’s primary sources of information except prenatal class, and influential persons’ impact on continued breastfeeding to determine which factors were significantly associated with continued breastfeeding at one year postpartum. All statistical analyses were performed with SPSS for Windows version 22 (IBM Corp., Armonk, NY, USA), and a *p*-value of equal to or less than 0.05 was considered statistically significant.

## 3. Results

### 3.1. Participant Characteristics

The questionnaire was completed by 451 mothers whose child was between 12 and 24 months old, with 57% of mothers reporting breastfeeding at 12 months. The main characteristics of the mothers are described in Table 1. The mean age (±SD) of mothers was 30.97 ± (5.31) years, and 52.8% were older than thirty years. The mean age (±SD) of the children was 16.11 ± (3.44) months, and 73.2% were between 12 and 18 months old (data not shown). Nearly all the participants were married (97.1%). Two-thirds of mothers earned a household income less than 950 USD per month (68.1%) and were working mother (62.1%). In addition, approximately half of mothers had an undergraduate level of education (54.1%). The majority of mothers who successfully continued breastfeeding at one year postpartum had a graduate-level education (62.3%, *p* = 0.035) and were multiparous (63.4%, *p* = 0.037) (Table 1).

### 3.2. Factors Encouraging the Decision to Breastfeed

**Figure 1:**
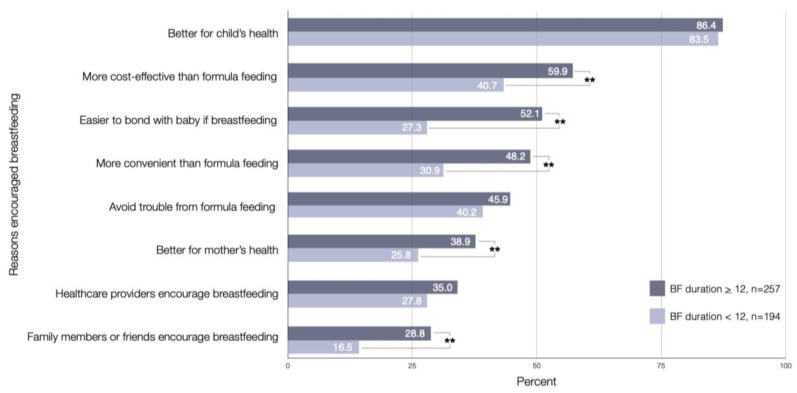
Factors encouraging breastfeeding as identified by mothers who continued and not continued breastfeeding at one year postpartum (*n* = 257, *n* = 194); ** significant association at *p* < 0.01.

The mothers who successfully continued breastfeeding at one year postpartum (*n* = 257) described the reasons that influenced their breastfeeding maintenance for 12 months, as illustrated in Figure 1. The most frequent reason identified by mothers who success continued breastfeeding at one year postpartum was that it was better for children’s health (86.4%). A second and third main reasons were that breastfeeding is more cost-effective than formula feeding (59.9%), and breastfeeding makes it easier to bond with the baby (52.1%). There was a significant association between all reasons that influenced their breastfeeding maintenance at one year postpartum except better for child health, avoid trouble from formular feeding and healthcare providers encourage breastfeeding. 

### 3.3. Mother’s Primary Sources of Information Related to Continued Breastfeeding at One Year Postpartum

**Figure 2:**
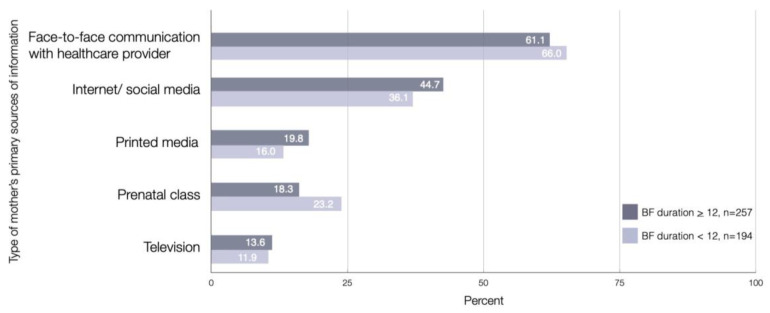
Mother’s primary sources of information were related to continued and not continued breastfeeding at one year postpartum (*n* = 257, *n* = 194).

The majority of mothers who continued breastfeeding at one year postpartum reported that face-to-face communication with healthcare providers (61.1%) and internet and social media (44.7%) were the important communication types that influenced breastfeeding practice at one year. In comparison, only one-fifth of the participants received information related to extended breastfeeding from printed media (19.8%) and prenatal class conferences (18.3%). Television was less often reported as the source of information (13.6%). There was no significant association between all mother’s primary sources of information and continued breastfeeding at one year postpartum (Figure 2).

### 3.4. Influential Persons Impacting Continued Breastfeeding

**Figure 3:**
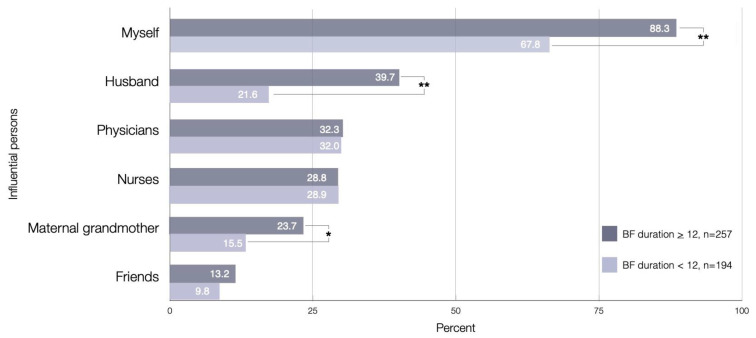
Influential persons who impacted the decision to continue breastfeeding, as identified by mothers who continued and not continued breastfeeding at one year postpartum (*n* = 257, *n* = 194); * significant association at *p* < 0.05; ** significant association at *p* < 0.01.

Regarding the important person who was influential in the decision to breastfeed for at least 12 months, the most frequent answer was ‘myself’ (88.3%). The husband (39.7%) was the second most important person who influenced the decision to continue breastfeeding. Health professionals, including physicians (32.3%) and nurses (28.8%), were less frequently reported. There was a significant association between the influential persons impacting continued breastfeeding as myself, husband, and maternal grandmother with continued breastfeeding at one year postpartum (Figure 3).

### 3.5. Predictors of Continued Breastfeeding at One Year Postpartum

Binary logistic regression was conducted to identify the factors associated with continued breastfeeding at one year postpartum. Maternal graduate education, multiparous status, full-time work with day shift, work-from-home job, and factors encouraging breastfeeding as easier to bond with baby were found to be associated with continued breastfeeding at one year postpartum (*p* < 0.05) (Table 2).

A multivariate logistic regression model showed that mothers with undergraduate education (AOR = 0.635; 95% CI: 0.404, 0.997, *p* = 0.049), had lower odds of continued breastfeeding than graduate while multiparous status (AOR = 1.588; 95% CI: 1.042, 2.420, *p* = 0.031), had higher odds of continued breastfeeding than primiparous. In addition, our result showed that working was a negative predictor of breastfeeding outcomes. Working mothers with various working schedules, including full-time work with a day shift (AOR = 0.437; 95% CI: 0.261, 0.731, *p* = 0.002, work with a rotational shift (AOR = 0.481; 95% CI: 0.247, 0.934, *p* = 0.031), and work-from-home jobs with a flexible schedule (AOR = 0.439; 95% CI: 0.229, 0.838, *p* = 0.013), had lower odds of continued breastfeeding than SAHMs. Considering, mothers described it being easier to bond with their babies when breastfeeding as a reason contributing to continued breastfeeding had higher odds of continuing breastfeeding than their counterparts (AOR = 3.118; 95% CI: 2.022, 4.809, *p* < 0.001). Nevertheless, another type of communication, influential person, and other maternal characteristic variables did not show statistically significant associations with continued breastfeeding at one year (Table 2).

## 4. Discussion

We reported maternal and external factors affecting continued breastfeeding at one year among women who visited the well-baby clinic in secondary and tertiary hospitals in Chiang Mai, Thailand. Our main finding was that reporting “easier to bond with my baby if breastfeeding “as a reason to continue breastfeeding and multiparous status were positive predictors, while undergraduate educational level and working were negative predictors. The percentage of mothers in our sample who continued breastfeeding at 12 months (57%) was higher than the national data (24.6%) from a recent national survey in 2019 [20]. This difference was probably due to the recruitment process, which was limited to populations in the baby-friendly hospital initiative that may have led to a higher success rate of breastfeeding at one year. 

Easier to born with baby, better for mother health, encourage by family members or friends, and breastfeeding have an advantage over formula feeding (cost effective and convenient) were important reasons as identified by mothers who success continued breastfeeding at one year postpartum. In multiple regression analysis, we identified that the only factor that was a strong predictor of breastfeeding at one year was “Easier to bond with baby if breastfeeding”. Most of the earlier studies explored attitudes toward exclusive breastfeeding at six months and found that the mother frequently mentioned that breastfeeding improves bonding between the mother and child [36,37]. It is difficult to compare our results with those of a prior study because the reason for encouraging breastfeeding, as identified by mothers who continued breastfeeding at one year postpartum, has not been identified before. Looking at the results of a limited study that identified the attitude of mothers who continued breastfeeding for over one year, long-term breastfeeding rates were higher in mothers who had favorable breastfeeding attitudes [25] and prenatal intentions to breastfeed [23].

Conversations with healthcare professionals and the internet or social media were the common primary sources of information reported by 60% and 45% of participants who success breastfeeding for at least 12 months, respectively. In contrast, less than one-five of the mothers reported other mass media, including television or printed media, as sources of information. In Thailand, 98% of families have television service [38], and approximately 75% of the total population are active internet users [39]. Although television is the generally accessible mass media in Thailand, the internet or social media are the most common types of mass media from which our participants gained information related to prolonged breastfeeding. This finding is consistent with recent research, which has found that online support groups are a relatively new trend in Thailand, and Facebook has become a platform for Thai parents to seek informational and emotional support related to child feeding content [40]. Apart from health care professionals, husbands and maternal grandmothers were more frequently mentioned as influential persons with extended breastfeeding than friends. A similar finding was observed in many other studies; family members’ attitudes toward breastfeeding positively impacted breastfeeding duration [21,27,28,41,42]. The information presented by media can increase the knowledge and shape attitudes, norms, and behaviors related to breastfeeding [42,43]. To raise prolonged breastfeeding awareness, we suggest that both lactating mothers and their family members should be informed through the internet or social media networks together with a conventional promotional approach by the healthcare provider.

Multivariable logistic regression analysis was used to investigate the relationship between explanatory variables and continued breastfeeding. Our results highlighted that working mothers participating in any shiftwork type, including full-time work with day shifts, work with rotational shifts, and work-from-home jobs with flexible schedules, by the time their child was 12 months old were less likely to continue breastfeeding than SAHMs. Most previous research has focused on infant age when returning to work and did not explore the type of work. Mothers who returned to work at the end of the first year were significantly less likely to breastfeed until 12 months was reported by previous articles [28,29,31]. A large birth cohort study in Southwestern Sydney, Australia, examined maternal employment status at 12 months, including full-time employment, part-time employment, casual employment and no return to work. Compared to unemployed mothers, mothers with full-time employment or part-time employment had a significantly higher risk of ceasing breastfeeding at 12 months. However, this study shows no association between casual employment and the duration of lactation [27]. The negative impact of workplace on breastfeeding practices was not surprising because physical and social environments in the workplace may be the barrier to continue breastfeeding, such as the lack of dedicated space, inflexible work schedules, inadequate facility in which to pump and to store milk, and stress from work [44,45]. In addition, we identified work-from-home jobs with a flexible schedule as a factor associated with the risk of stopping breastfeeding before 12 months. The negative association between working from home and breastfeeding may seem unexpected because the mother can provide breast milk to their babies without the barriers associated with the workplace mentioned above. However, when working from home, mothers have to handle particular issues, such as irregular breaks, irregular video calls, or distraction related to household chores. Therefore, maintaining a nursing schedule may be a challenge for work-from-home mothers.

The results of this study observed that undergraduate mothers are a potential risk factor for discontinuing breastfeeding before one year postpartum. This is consistent with previous research which identified the association between maternal education and breastfeeding duration [26,27,28]. A recent study in Australia identified the association between maternal education and breastfeeding duration; mothers who had completed a university degree had a 47% (AHR = 0.53) lower risk of stopping breastfeeding at 12 months than mothers who did not complete high school [27]. Parity was the common factor that was tested for its association with the maintenance of breastfeeding [46]. The experience acquired from previous children may positively influence breastfeeding duration because women who have experienced motherhood before and have more knowledge that may help them overcome any difficulties during breastfeeding. Multiparous women were more likely to initiate breastfeeding early and to exclusively breastfeed for 6 months than primiparous mothers [47,48,49]. Moreover, the positive association of parity on breastfeeding at 12 months was reported in this study and others [28,30]. Nor did we find an association between the maintenance of breastfeeding for 12 months and maternal age, maternal income, the timing of first antenatal care, and infant age when mother returned to work, which may deference result from the various populations studied in terms of effect and magnitude [46]. 

Based on the major findings of this study, working mothers both employed outside the home and working from home were at risk of discontinuing breastfeeding before 12 months. Creating workplace breastfeeding-support programs, including establishing dedicated breastfeeding rooms, facilities for expressing and storing breast milk, breastfeeding breaks, or creating on-site childcare may help formally employed mothers breastfeed their babies longer if they choose [45,50]. In addition, future research should explore the barriers associated with working from home, and the results can be implemented in new policies to support work-from-home mothers who lack regulation or benefit from legal protection.

To the best of our knowledge, this is the first work that focuses on the factors influencing continued breastfeeding at 12 months in Thailand. The strengths of our study are that the data collection was performed in secondary and tertiary public hospitals and private hospitals, providing access to a wide range of patients of various socioeconomic statuses. Moreover, the data were collected by three trained researchers who conducted face-to-face interviews. This study has some limitations that must be considered when interpreting the findings. First, this study has the limitations of a cross-sectional study design. Second, information on perinatal care and breastfeeding practice was obtained at 12 and 24 months after birth; therefore, there is a possibility of recall bias. Third, the data collection was performed in secondary and tertiary hospitals which implemented a baby-friendly hospital initiative; thus, applying this study’s findings to the general population would require further investigation.

## 5. Conclusions

This study reports the factors affecting continued breastfeeding at one year among women who visited the well-baby clinic in secondary and tertiary hospitals in Chiang Mai, Thailand. Reporting “easier to bond with baby” as a reason to continue breastfeeding and multiparous status were positive predictors, while mothers with undergraduate education levels were more likely to ceasing breastfeeding before one year. Maternal employment status, including both employed outside the home and work from home with any shiftwork types, was a risk factor for discontinuing breastfeeding before 12 months. To enhance the continuance of breastfeeding, policies promoting supportive breastfeeding in the workplace should be implemented. Work-from-home mothers and women who did not reach the 12-month breastfeeding target require more research to explore the related barriers and establish more support strategies. Additionally, a longitudinal approach to breastfeeding research in the general population could enrich data and be an important area for future research.

## Figures and Tables

**Table 1:** Demographic and maternal characteristics (*n* = 451).

Characteristics	Total (*n* = 451)	The Duration of Breastfeeding, *n* (%)		*p*-Value
		BF ≥ 12 Months (*n* = 257, 57.0%)	BF < 12 Months (*n* = 194, 43.0%)	
Maternal age at birth (years); Mean ± SD	30.97 ± 5.31	31.16 ± 5.32	30.71 ± 5.29	
≤25	74 (16.4)	40 (54.1)	34 (45.9)	0.836
26–30	139 (30.8)	79 (56.8)	60 (43.2)	
>30	238 (52.8)	138 (58.0)	100 (42.0)	
Level of education				
Undergraduate	244 (54.1)	128 (52.5)	116 (47.5)	0.035 *
Graduate ^a^	207 (45.9)	129 (62.3)	78 (37.7)	
Monthly income of the household, USD ^¥^				
≤950	307 (68.1)	171 (55.7)	136 (44.3)	0.643
950–1900	101 (22.4)	59 (58.4)	42 (41.6)	
>1900	43 (9.5)	27 (62.8)	16 (37.2)	
Marital status				
Married	438 (97.1)	252 (57.5)	186 (42.5)	0.139
Other (Single/Window/Separated)	13 (2.9)	5 (38.5)	8 (61.5)	
Body Mass Index (BMI), kg/m^2^				
Underweight (<18.5)	45 (10.2)	27 (60.0)	18 (40.0)	0.727
Normal weight (18.5–22.9)	213 (48.1)	122 (57.3)	91 (42.7)	
Overweight (23.0–24.9)	79 (17.8)	44 (55.7)	35 (44.3)	
Class 1 obesity (25.0–29.9)	80 (18.1)	49 (61.2)	31 (38.8)	
Class 2 obesity (≥30.0)	26 (5.9)	12 (46.2)	14 (53.8)	
Parity				
Primiparous	287 (63.6)	153 (53.3)	134 (46.7)	0.037 *
Multiparous	164 (36.4)	104 (63.4)	60 (36.6)	
Timing of first antenatal care (ANC)				
First trimester	379 (84.0)	214 (56.5)	165 (43.5)	0.849
Second trimester	56 (12.4)	33 (58.9)	23 (41.1)	
Third trimester	16 (3.5)	10 (62.5)	6 (37.5)	
Age of infant when mother returned to work, months				
≤3	376 (83.7)	219 (58.2)	157 (41.8)	0.232
>3	73 (16.3)	37 (50.7)	36 (49.3)	
Shiftwork types				
Stay-at-home mothers (SAHMs)	170 (37.9)	109 (64.1)	61 (35.9)	0.096
Full-time work with day shift	164 (36.5)	87 (53.0)	77 (47.0)	
Work with rotational shift	59 (13.1)	33 (55.9)	26 (44.1)	
Work-from-home job with a flexible schedule	56 (12.5)	27 (48.2)	29 (51.8)	

Analyzed by chi-square test, ^a^ college degree or higher education, ^¥^ = 1.00 USD = 32.00 THB, BF = Breastfeeding, * significant association at *p* < 0.05.

**Table 2:** The association between factors and continued breastfeeding at one year postpartum (*n* = 451).

Variables	Continued BF at One Year Postpartum			
	^a^ Crude OR (95% CI)	*p*-Value	^b^ Adjusted OR (95% CI)	*p*-Value
Maternal education				
Undergraduate	0.667 (0.458–0.973)	0.035 *	0.635 (0.404, 0.997)	0.049 *
Graduate	1 (Reference)		1 (Reference)	
Parity				
Primiparous	1 (Reference)		1 (Reference)	
Multiparous	1.518 (1.024, 2.250)	0.038 *	1.588 (1.042, 2.420)	0.031 *
Shiftwork types				
Stay-at-home mother (SAHM)	1 (Reference)		1 (Reference)	
Full-time work with day shift	0.632 (0.408, 0.980)	0.040 *	0.437 (0.261, 0.731)	0.002 **
Work with rotational shift	0.710 (0.389, 1.297)	0.265	0.481 (0.247, 0.934)	0.031 *
Work-from-home job with a flexible schedule	0.521 (0.283, 0.960)	0.036 *	0.439 (0.229, 0.838)	0.013 *
Factors encouraging the decision to breastfeed				
Easier to bond with baby if breastfeeding	2.898 (1.944, 4.322)	<0.001 **	3.118 (2.022, 4.809)	<0.001 **

^a^ analyzed by binary logistic regression; ^b^ analyzed by multiple logistic regression with forward stepwise method; BF = breastfeeding; OR = odds ratio; * significant association at *p* < 0.05; ** significant association at *p* < 0.01.

## Data Availability

The data presented in this study are available on request from the corresponding author.
